# Redescription and anatomy of *Diplodonta portesiana* (d’Orbigny, 1846) (Bivalvia, Ungulinidae) from Brazil

**DOI:** 10.3897/zookeys.275.3782

**Published:** 2013-03-04

**Authors:** Bárbara L. V. Romera, Luiz R. L. Simone, Carlo M. Cunha

**Affiliations:** 1 Museu de Zoologia da Universidade de São Paulo. Laboratório de Malacologia. Avenida Nazaré, 481. CEP: 04263-000, Brazil

**Keywords:** *Diplodonta portesiana*, Ungulinidae, anatomy, taxonomy, Atlantic Ocean

## Abstract

The present redescription of *Diplodonta portesiana* (d’Orbigny, 1846) is the first part of the revision of this genus in the East Atlantic. This species, despite being common in the Atlantic coast, remains poorly known. A detailed shell and anatomical study was conducted based not only on specimens from the type locality’s vicinities but also on samples from other regions. Diagnostic characters for *Diplodonta portesiana* includes: rounded shell with a small ligament; triangular, short and deep nymph; external micro ornamentation composed of small concavities in a concentric pattern; small adductor muscles; reduced pedal gape; pair of long hemipalps with a large area covered by folds; stomach with four ducts leading to digestive diverticula; and long intestine length. Our study suggests at least two new diagnostic characters to the genus: the two pair of muscles that controls the incurrent and excurrent openings and a residual ring-like tissue surrounding the anterior half of the posterior foot retractor muscle.

## Introduction

The ungulinid genus *Diplodonta* Bronn, 1831 [type: *Venus lupinus* Broochi, 1814; Italy, Piemonte, Andona Valey; subsequent designation by Bronn 1831] shows a nearly worldwide distribution (Olsson 1961; Abbott 1974; Coan et al. 2000; Redfern 2001; Mikkelsen and Bieler 2007; Coan and Valentich-Scott 2012). Despite being especially common in the South East Atlantic coast, and as was already noted by Dall (1901), the genus’ taxonomy remains problematic, with high oscillation of number of species. Recent shell catalogues frequently show, in our concept, incomplete character lists and descriptions (e.g., Rios 2009). The last revision is half a century old and dealt only with the genus’ diagnostic characters (Chavan 1962). Moreover, the last detailed revision of the West Atlantic species complex was conducted almost one century ago (Lamy 1920), and it barely elucidates the species identification, only grouping names that were created in previous revisions, apparently lacking any discernment. The single detailed anatomical study including the genus *Diplodonta* (Allen 1958) is a comparison among members of the superfamily Lucinoidea, the superfamily used to include the Ungulinidae before recent revision (Williams et al. 2004).

The present paper is the first of a series concerned with the systematic revision of the West Atlantic *Diplodonta*. It focuses on *Diplodonta portesiana* (d’Orbigny, 1846), a common but usually misidentified regional species. The present redescription is based on type specimens and topotypes with a wide bulk of samples, and also extends the species’ definition to include its anatomy.

## Material and methods

A complete list of examined material is presented after the description. The holotype was examined and the other ~40 samples contain individuals from several localities. Shell measures were taken with a digital caliper: length corresponds to the greatest distance between anterior and posterior margin; height is the longest distance between the dorsal umbo’s end and the ventral margin; width is the greatest straight line reaching from the right to the left valve. The dissected specimens were fixed in 70% ethanol and studied under a stereomicroscope by standard techniques (Simone 2009). All drawings were made with the aid of a camera lucida. The Scanning Electron Microscopy (SEM) was provided by the Laboratório de Microscopia Eletrônica do Instituto de Biociências from the Universidade de São Paulo.

The following abbreviations are used in the anatomical descriptions and figures: **aa:** anterior adductor muscle; **an:** anus; **cc:** cerebral connective; **cg:** cerebral ganglia; **cn:** ctenidial nerve; **cp:** cerebro-pedal connective; **cv:** cerebro-visceral connective; **dd:** digestive diverticula; **dg:** digestive gland; **dh:** dorsal hood; **em:** excurrent muscular wall; **er:** esophageal rim; **es:** esophagus; **fp:** posterior foot retractor muscle; **fr:** anterior foot retractor muscle; **ft:** foot; **gi:** gill; **go:** gonad; **gs:** gastric shield; **gt:** gill tissue; **he:** heart; **id:** inner demibranch; **im:** pair of incurrent channel muscle; **in:** intestine; **ip:** inner palp; **ki:** kidney; **lp:** labial palp; **ld:** left diverticula; **lp**: left pouch; **mt:** major typhlosole; **na:** anterior adductor muscle nerve; **np:** nephropore; **nt:** minor typhlosole; **od:** outer demibranch; **op:** outer palp; **pa:** posterior adductor muscle; **pg:** pedal ganglia; **pm:** pallial muscle; **pn:** pallial nerve; **rd:** right diverticula; **rn:** renal nerve; **sm:** pair of excurrent channel muscle; **ss:** style sac; **st:** stomach; **vg:** visceral ganglia; **vm:** visceral mass.

Institutional abbreviations: **CENEMAR**, Centro de Estudos Marinhos do Atlântico Sul, Porto Alegre, Brazil; **CMAC**, Museo Argentino Del Caracol, Argentina; **MZSP**, Museu de Zoologia da Universidade de São Paulo, Brazil; **MZUCR**, Museu de Zoologia da Costa Rica, Costa Rica; **NHMUK**, Natural History Museum of United Kingdom.

## Systematics

### Diplodonta portesiana

(d’Orbigny, 1846)

http://species-id.net/wiki/Diplodonta_portesiana

**Figures 1–6.**
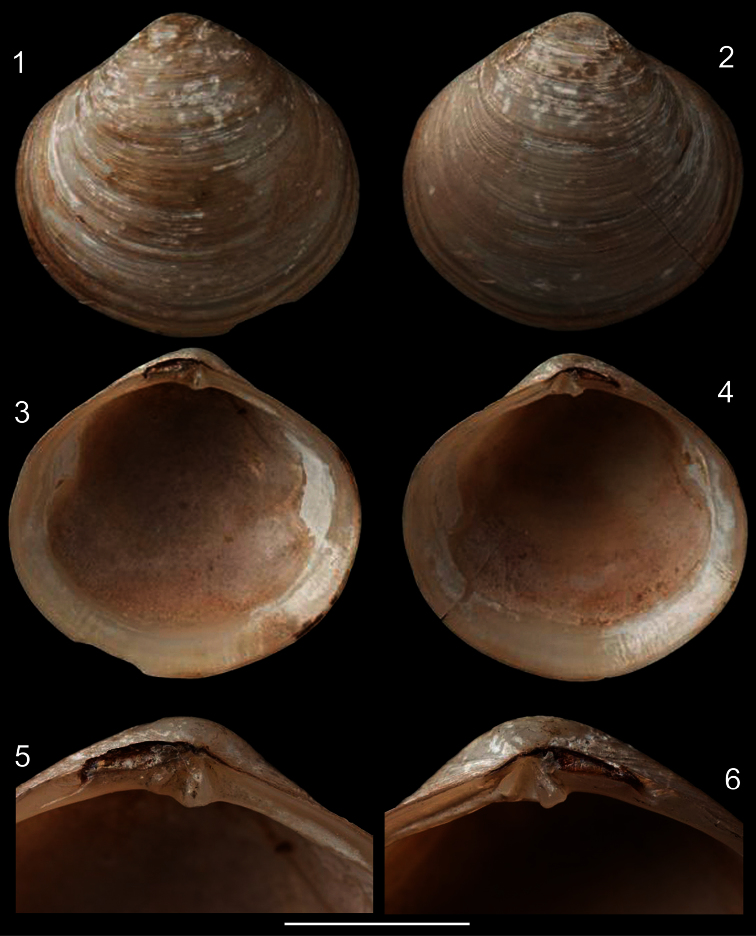
*Diplodonta portesiana*. Holotype (NHMUK 1854.12.4.770; L: 20 mm; H: 17 mm). **1** Left valve, external view **2** Right valve, external view **3** Left valve, internal view **4** Right valve, internal view **5** Left hinge detail **6** Right hinge detail. Scale: 2 mm.

Figs 1 [Fig F2] [Fig F3] –29

Lucina portesiana d’Orbigny, 1846: 586 (pl. 81, Figs 12–13); Gray 1857: 72; Baril 1862: 137; Tryon 1872: 84; Aguirre 1994: 357 (pl. 2, Figs 17a, b).
Diplodonta portesiana : Dall 1901: 794; Amaral et al. 1999: 44; 2010: 238.
Diplodonta nucleiformis : Morris 1947: 44 (pl. 20, fig. 22); Warmke and Abbott 1962: 175 (pl. 35, fig. j); Rios 1970: 174; 1975: 218 (fig. 1049); 1985: 233 (fig. 1165); 1994: 255 (fig. 1247); 2009: 518 (fig. 1440) (non Wagner 1838).

#### Type.

Holotype NHMUK 1854.12.4.770 (Figs 1–6) (examined).

#### Type locality.

São Cristóvão Bay, Rio de Janeiro, Brazil.

#### Redescription.

**Figures 7–14. F2:**
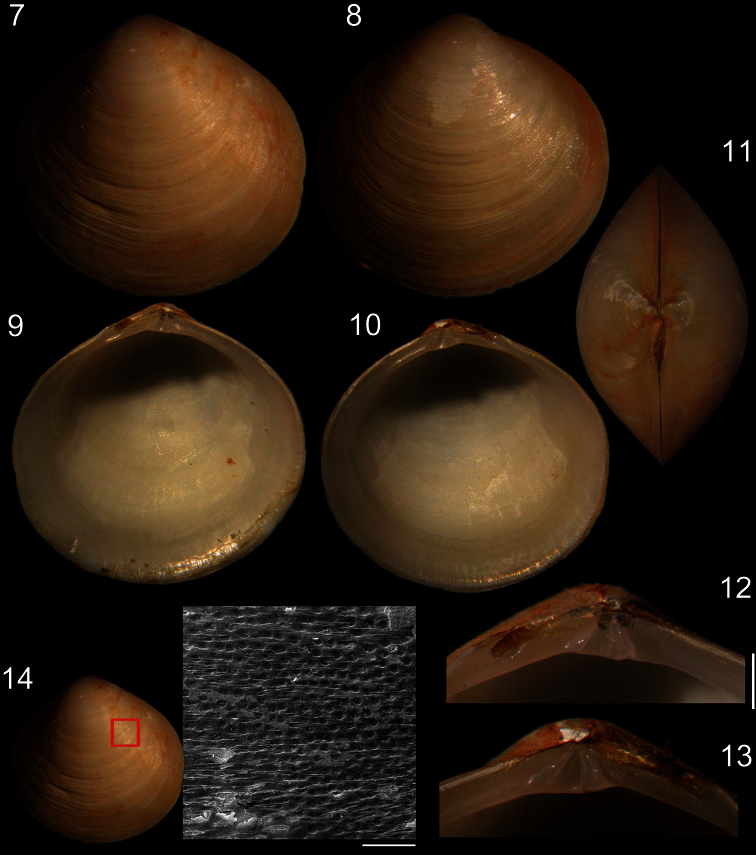
Specimen of *Diplodonta portesiana* (MZSP 22747, L: 13.4 mm, H: 13.1 mm, width: 7.7 mm). **7** Left valve, external view **8** Right valve, external view **9** Left valve, internal view **10** Right valve, internal view **11** Dorsal view **12** Left hinge detail **13** Right hinge detail **14** External surface of shell under SEM; Scale: 2 mm, except **14**: 200 µm.

*Shell* (Figs 1–14): Rounded, centrally pointed, equivalve and inequilateral. Laterally inflated (Fig. 11), maximum inflation ~60% of total length. Externally covered by shallow concentric ribs. Pattern of horizontal aligned, small, rounded pits, slight parallel to concentric growth lines, only visible under SEM (Fig. 14). Color white, periostracum thin, translucent, rarely cream to dark brown close to edges. Valves fragile; internally opaque (Figs 9–10). Anterior adductor muscle scar reniform, ventral portion 2.5 times wider than dorsal portion; located between mid and dorsal thirds of shell height. Posterior adductor muscle scar elliptical, same distance from shell border as anterior adductor, located in median third of valve height. Pallial line entire and thin, away from shell border ~10% of valve height. Umbos low, ~5% of total shell height. Ligament parvincular, opisthodetic, ~20% of total shell length (Figs 11–13). Hinge heterodont, with two cardinal teeth, anterior tooth of left valve and posterior tooth of right valve bifid (Figs 12, 13). Dental shelf long, length equivalent to half of total dorsal margin length, close to cardinal teeth. Dorsal margin concave, forming groove at fusion with dental shelf. Lateral teeth absent. Nymph short, ~20% of total shell length, ~5 times wider than long, triangular. Lunule and escutcheon absent.

**Figures 15–22. F3:**
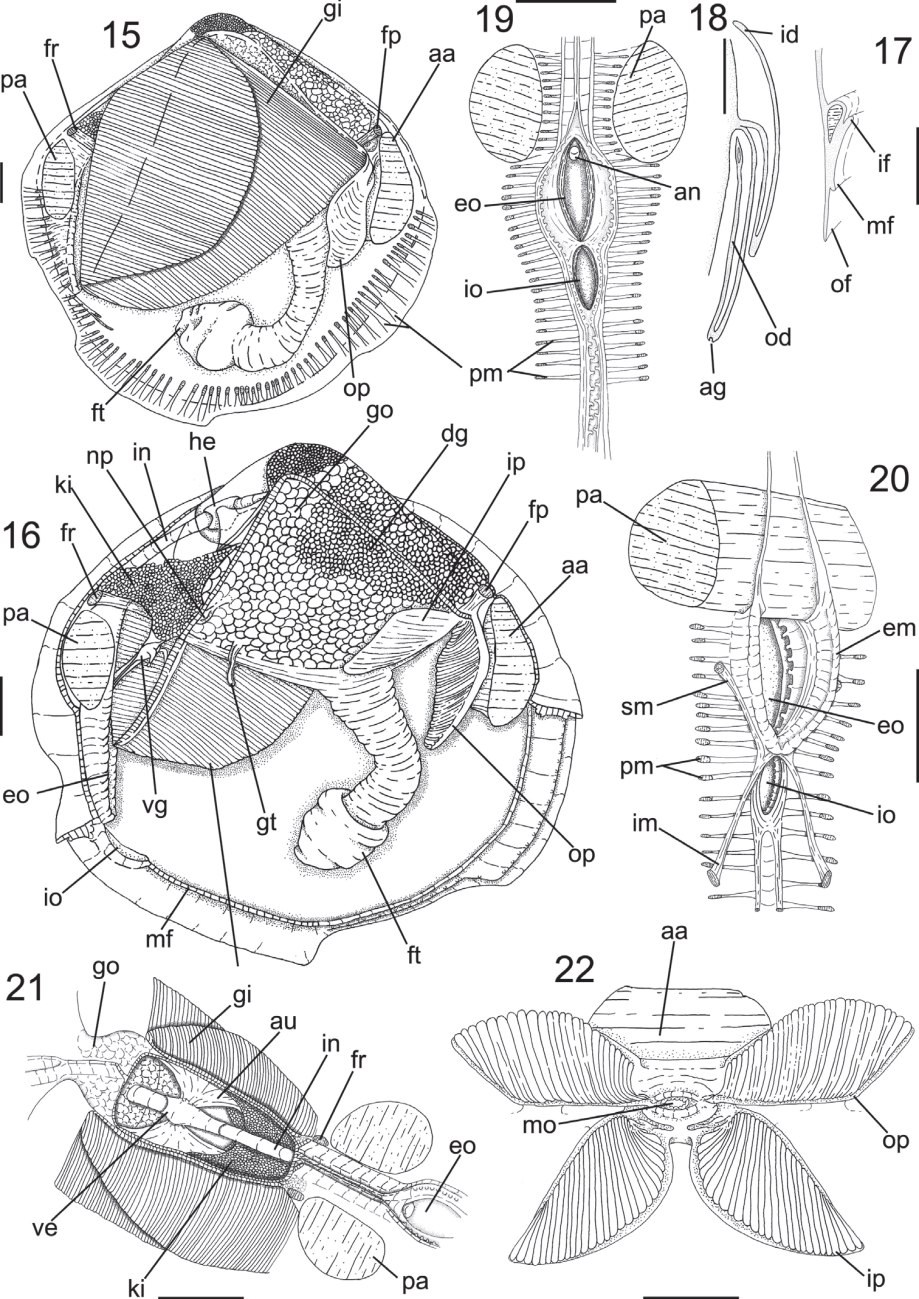
Anatomy of *Diplodonta portesiana*. **15** Right view, right mantle lobe removed **16** Same view, gill removed **17** Mantle border, transverse section in median portion **18** Gill, transverse section in middle portion **19** Incurrent and excurrent openings, posterior view **20** Same, anterior-slightly right view **21** Pericardium region, dorsal view **22** Labial palps, ventral view, hemipalps deflected; Scale: 2 mm, except **17**, **18**: 1mm.

**Figures 23–29.**
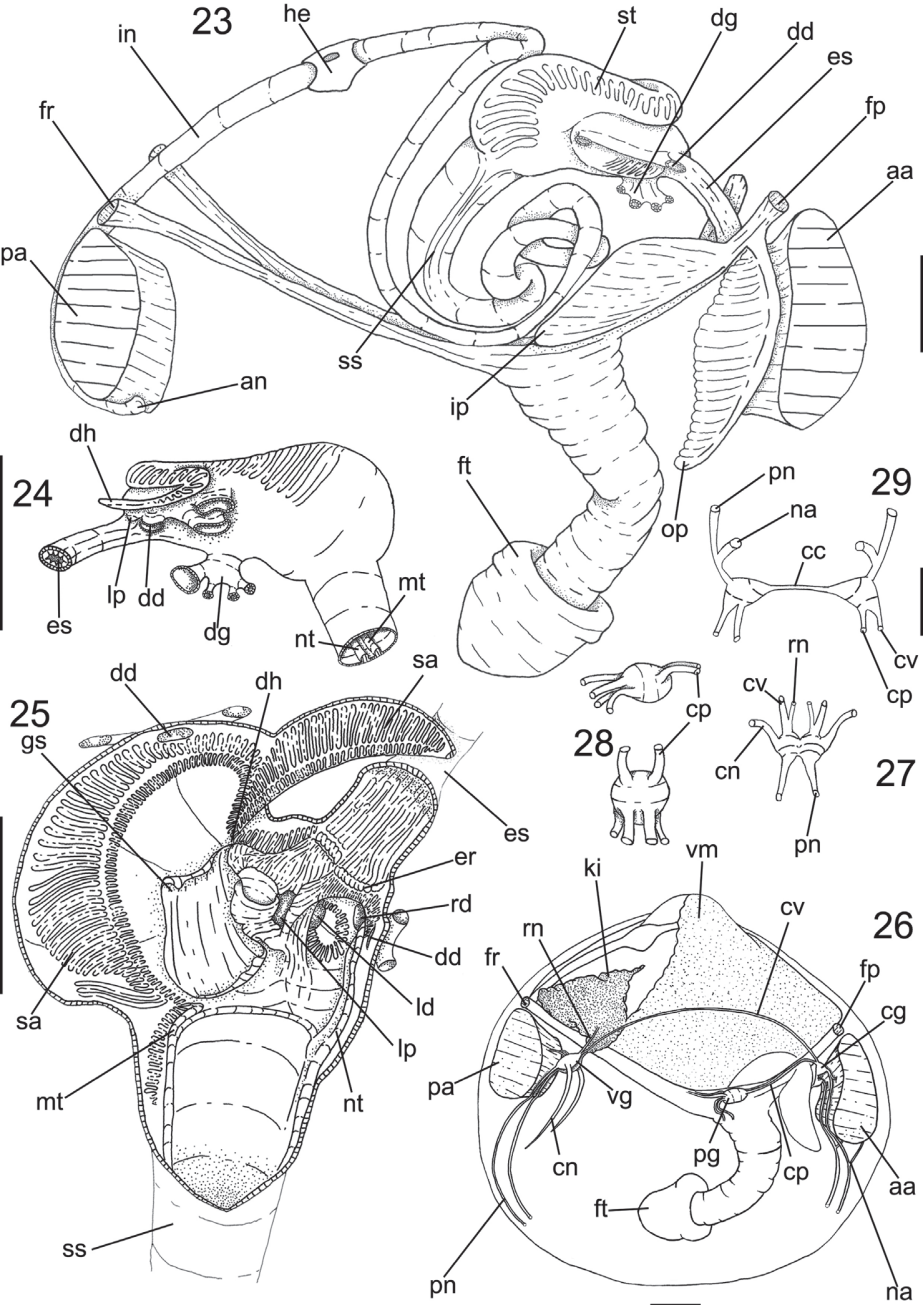
Anatomy of *Diplodonta portesiana*. **23** Digestive system as in situ, right view, foot and main muscles also shown **24** Stomach, left view **25** Internal stomach surface, right view, dorsal gastric wall sectioned longitudinally and deflected **26** Nervous system and topology of other main structures, right view **27** Visceral ganglia, ventral view **28** Pedal ganglia, superior figure in right view, inferior figure in ventral view **29** Cerebral ganglia, anterior view; Scale: **23, 26**: 2 mm; **24, 25**: 1 mm; **27–29**: 0,5 mm.

*Main muscles* (Figs 15, 16, 20, 23): Anterior adductor muscle reniform in section, ~2.6 times higher than wider, ventral half ~2.5 wider than dorsal half (Figs 15, 16, 23); occupying ~1/15 of internal shell volume, located between middle and dorsal third of shell height (Figs 15, 16, 20). Posterior adductor muscle ~40% smaller than anterior muscle, located at opposite end and parallel to anterior muscle (Figs 15, 16, 23). Pair of anterior foot retractor muscles oval in section and thin, originating dorso-posteriorly to anterior adductor muscle, insertion area ~1/20 of adductor insertion, length ~1/4 of total shell length, fusion of both branches occurring in its half-length (Figs 16, 23). Foot retractor muscles oval in section, slightly laterally compressed, thin, ~45% longer than anterior foot retractor muscles, inserting dorsally to anterior adductor muscle, in area equivalent to ~1/40 of anterior adductor insertion, both branches fusing in ventral quarter of shell length (Figs 16, 23). Incurrent and excurrent openings surrounded by two pairs of muscles (Fig. 20); pair of incurrent channel muscles bordering incurrent opening, inserting at dorsal ending of opening; long, straight and thin, equivalent to twice opening length (Fig. 20: im); pair of excurrent channel muscles narrow and thin, inserting below ventral ending of opening; bordering ~60% of excurrent opening length, ~30% shorter than pair of incurrent channel muscles (Fig. 20: sm).

*Foot and byssus* (Figs 15, 16, 23): Foot long, length ~70% of shell high; terminal bulb cylindrical, expanding ~30% beyond foot width, color orange to light brown; byssal groove and byssus absent.

*Mantle* (Figs 15–17): Mantle lobes symmetrical, thin, translucent, colorless. Pallial muscles strong and short, distributed evenly in ventral side of mantle lobe, length ~1/10 animal height (Fig. 15). Mantle border with three folds (Fig. 17): outer fold long and thin, ~1/3 of shell thickness, ~15 times higher than wide; middle fold short, twice wider and ~7.5 times shorter than outer fold; inner fold short, with twice as wide and ~5 times shorter than outer fold. Periostracum produced between outer and middle fold. Middle fold with 60 pairs of papillae, bordering all ventral border (Fig. 16); papilla twice taller than wide, tip rounded, separated by area equivalent to 5 times each papilla width. Mantle lobes partially free, fused in ~35% of total mantle lobes length, by inner fold; from incurrent opening to middle portion of animal’s length (Fig. 16: mf); free portion extending until ventral surface of ventral half of anterior adductor muscle, forming pedal gape, corresponding to ~55% of total shell length. Incurrent and excurrent openings formed of fusion of third mantle fold; flanked by ~30 pairs of papillae (Fig. 19: eo); excurrent opening ~1/4 of total shell length; incurrent opening ~60% of total excurrent opening length. No well-developed siphons; except for thick muscular wall internally at excurrent opening, acting like socket to demibranchs (Fig. 20: em). Small striped “ring like” tissue surrounding middle portion of posterior foot retractor muscle, below gill insertion at visceral mass (Fig. 16: gt).

*Pallial cavity* (Figs 15–16, 18, 22): Occupying half of inner shell volume (Fig. 16). Labial palps small, ~1/30 of inner shell area, triangular; external surface smooth; outer and inner hemipalps similar in size, ~7% longer and ~60% wider than anterior adductor muscle insertion area (Figs 16, 22); outer hemipalp connected to mantle lobe by dorsal border, in ~2/5 of total length; inner hemipalp connected to visceral mass by dorsal border, in ~1/4 of this total length; internal surface of both hemipalps covered by 30 transversal folds; outer hemipalp folds high and rounded in section, covering ~95% of surface, thin smooth area in dorsal margin, corresponding to 1/25 of total surface of hemipalp; folds of inner hemipalp flattened in section and centrally grooved in length; covering ~80% of internal surface of hemipalp, forming two thin smooth areas at ventral and dorsal borders, corresponding 1/8 of total surface of hemipalp; near mouth folds decreasing until forming small grooves converging at middle region of palps, towards to mouth (Fig. 22). Gills large, area ~16 times wider than outer hemipalp area, ~1/3 of total valve area (Figs 16, 18); with two demibranchs; outer demibranch fusiform, twice longer than wide; folded upon ~1/3 of own total area; covering pericardium and kidney; connected to mantle lobe by ~15% of length of postero-dorsal border; inner demibranch triangular, ~3 times longer than wide; folded upon half of total demibranch area; ~45% of external surface covered by outer demibranch; connection to visceral sac by cilia; gill filaments with rounded tips. Suprabranchial chamber volume ~3/5 of infrabranchial chamber volume (Fig. 16).

*Visceral mass* (Figs 16, 23): Visceral sac occupying half of inner shell volume; triangular, inflated, located dorsally between pair of anterior foot retractors and pair of posterior foot retractor, ~3 times larger than muscular base; ~35% of antero-dorsal region filled with greenish brown digestive gland, remainder areas filled with cream colored gonads. Stomach and style sac located vertically at central portion of visceral sac (Fig. 23).

*Circulatory and excretory systems* (Figs 16, 21): Pericardium located dorso-posteriorly of visceral sac, between umbonal cavity and dorsal kidney surface, occupying ~1/4 of visceral mass volume (Fig. 16). Pair of auricles antero-posteriorly elongated, connected to central axis of gills in 1/5 of gills total length; walls thick and translucent. Ventricle elongated, with thick walls; located at central portion of pericardium; surrounding ~1/3 of intestinal length crossing pericardium; connected to auricles at central portion of lateral walls (Fig. 21). Kidney located postero-ventrally in visceral mass (Fig. 16: ki), below posterior end of pericardium and dorsal surface of posterior foot retractor muscles, color light brown; shape triangular , occupying ~1/4 of visceral mass volume. Nephropore rounded, located at posterior portion of visceral mass, at ~10% of visceral mass height, opening in suprabranchial chamber (Fig. 16: np).

*Digestive system* (Figs 22–25): Palps and digestive glands described above. Mouth small, located centrally at palps intersection, with small lips (Fig. 22). Esophagus short and narrow, ~1/5 of total length of visceral sac and ~1/10 of high (Fig. 23), cylindrical; not touching anterior adductor muscle, crossing anterior third of anterior foot retractor muscles, parallel to anterior foot retractors (Fig. 23); inner surface covered by longitudinal folds until stomach entry, forming esophageal rim (Fig. 25); connected to anterior surface of stomach. Stomach of medium size, occupying ~1/5 of visceral sac volume, elliptical, located anteriorly at umbo; ~60% of visceral sac length and ~1/3 of high (Fig. 23: st); anterior portion ~30% wider than anterior portion. Pair of ducts to digestive diverticula located ventrally at anterior half of stomach, turned ventrally; digestive diverticula ducts connected to lateral walls of anterior half of stomach; one digestive diverticula duct connected at right wall, dorsally at esophagus line; one digestive diverticula duct connected at left wall, anteriorly to left pouch. Dorsal hood elongated and narrow, ~1/4 of stomach total length, ~4 times longer than wide, pointed anteriorly. Left pouch located below anterior ending of dorsal hood, anteriorly to digestive diverticula ducts; shallow and small, occupying area ~1/10 of surface of stomach (Fig. 24); internally anterior half of gastric chamber partially divided in ventral and dorsal portion by septum (Fig. 25); originating at anterior portion of right wall until dorsal hood entrance, septum length ~1/5 of total stomach length; dorsal surface covered by two sorting areas. Inner surface of stomach mostly smooth, with three distinct sorting areas; first sorting area starting at dorsal region of anterior portion, jointly to dorsal region of esophageal rim; heading dorsally until dorsal hood entrance and folding itself, covering posterior half of dorsal surface of gastric septum, narrow and long, composed of short and transverse folds with equivalent length and width; second sorting area starting on anterior half of dorsal surface of septum; heading from right side to dorsal hood, short and wide, composed of transverse folds twice longer than wide if compared with folds of first sorting area; third sorting area starting inside dorsal hood, running on latero-dorsally posterior right portion of stomach, entering style sac and attenuating. Gastric shield in central region of dorsal wall of stomach, occupying ~25% of total gastric area, translucent and iridescent, with two lateral projections, anterior projection penetrate left pouch and posterior projection penetrate dorsal hood; two gastric ridges narrow and tall running towards style sac, on ventral gastric surface, typhlosoles originating from each ridge. Long ridge originating in internal right wall of left digestive diverticula, leaving posteriorly and entering anteriorly in right side of left diverticula, forming semi-circle, touching left internal wall of diverticula and leaving anteriorly from ventral side of diverticula, on surface of anterior gastric portion, entering in anterior portion of right diverticula; bordering right lateral wall and leaving posteriorly, running on ventral wall of stomach until style sac opening, forming minor typhlosole (Fig. 25: nt) at ventral surface of sac. Small ridge running on ventro-posterior region of stomach, circular shape, flanking style sac entrance, running on ventral surface of sac forming major typhlosole (Fig. 25: mt). Style sac (ss) connecting ventrally to dorsal portion of stomach; conic; narrowing towards dorsal region of visceral sac, ~3.5 longer than wide; occupying ~1/6 of visceral sac total volume, height half and width ~1/10 of visceral sac high (Fig. 23). Intestine narrow and long, originating in level where typhlosoles decreasing in shape at internal surface of style sac (Fig. 23); running towards ventral portion of visceral sac, below central portion of stomach; with three overlapping loops, last one covering dorsally other two; following to posterior portion of visceral sac, parallel to style sac; forming loop on dorso-posterior surface of stomach, leaving visceral sac, crossing pericardium and posterior surface of kidney, crossing between pair posterior foot retractor muscles; touching entire posterior surface of posterior adductor muscle (Fig. 23); intestine total length ~10 times longer than style sac. Anus simple, sessile, opening at ventral region of posterior adductor muscle.

*Genital system* (Fig. 16): Gonads cream- colored, in granular aspect. Pair of gonoducts connected to gonad acini along posterior portion of visceral sac. Genital pore simple, located at posterior region of visceral sac, opening inside renal chamber.

*Central nervous system* (Figs 26–29): Pair of cerebral ganglia (cg) surrounding dorsal surface of anterior portion of esophagus (Fig. 29); dorsally at external surface of outer labial palp; triangular shaped, longer than wide; length half of esophagus length; each ganglion with ~1/3 of a transverse section of esophagus; cerebral commissure ~1/7 longer than ganglia total length; from anterior portion connecting anterior adductor muscle nerve (na), touching posterior surface of anterior adductor muscle, bifurcating in two branches; internal branch penetrating dorso posterior third of muscle and leaving at ventral surface of muscle; outer branch bordering posterior surface of anterior muscle, both branches fusing at ventral region of anterior muscle (Fig. 26); two connectives originating dorsally in ganglia, anteriorly to cerebro-visceral connective (cv) crossing visceral mass, touching gonopore dorsally, bordering anterior portion of kidney and connecting dorsally at visceral ganglia, connecting posteriorly cerebro-pedal connective (cp) running immersed in pedal muscles, connecting to anterior region of pedal ganglia. Pair of visceral ganglia (vg) fusiform, small, length same than height, ~1/2 of cerebral ganglia length, partially fused at median portion, with slightly central groove (Fig. 27); located ventrally to kidney, parallel to posterior adductor muscle, in dorsal tip connecting cerebra-visceral connective (described above) and renal nerve (rn), penetrating into kidney area; laterally originating ctenidial nerves (cn) running thought central axis of posterior portion of gills; dorsally originating posterior adductor muscle nerve, penetrating middle region of anterior surface of posterior muscle; at ventral tip originating pallial nerve (pn), touching anterior surface of ventral portion of posterior adductor muscle, running parallel to incurrent and excurrent apertures and mantle border, diffusing at mantle lobe board (Fig. 26). Pair of pedal ganglia (pg) oval, longer than wide, totally fused with each other, similar length and height of cerebral ganglia (Fig. 28); located internally to posterior retractor pedal muscles, above foot insertion, in anterior tip connecting cerebro-pedal connectives from cerebral ganglia; in posterior tip connecting two pairs of nerves, dorsal pair running towards posterior region, inside posterior foot retractor muscles; postero-ventral nerves curved to ventral region, running internally foot.

#### Habitat.

Infaunal, in muddy sands; from 0 to 68 m depth.

#### Measurements

(*length, height and width in mm*). MZSP 22747 (Figs 7–14): 13.4 by 7.1 by 7.7; MZSP 105725 #1: 16.4 by 16.1 by 10.1; CENEMAR: 17.6 by 17.3 by 11.7.

#### Geographic range.

From Limón, Cahuita, Costa Rica, to, Bombinhas, Santa Catarina, Brazil.

#### Material Examined.

Holotype HMUK 1854.12.4.770 (d’Orbigny col. 1842); COSTA RICA. **Limón**; Refugio Nacional de Vida Silvestre, Gandoca- Manzanillo, between Gandoca and Rio Sixaola river mouth, 12 m depth, 09°34'37.8966"N, 82°34'33.0309"W, MZUCR- INB0003816046, 3 valves (T: 181-S. Avilla/Lote: 73904, A. Berrocal id., S. Ávila col. 12/vi/2003); Cahuíta, National Park, 1 km to east of Puerto Vargas. 12 m depth, 9°43'33.3681"N, 82°48'31.5178"W, MZUCR- INB0003414093, 2 valves (S. Ávila id, S. Ávila col. 09/vi/20010); 3 km to North east of Cahuita, reef out, 12 m depth. 9°45'50.3530"N, 82°49'29.9092"W, MZUCR- INB0003411964, 2 valves, (S. Ávila id., J. Magaña col. 11/vi/2001); Punta Cahuita, 8 m depth, 9°44'58.2370"N, 82°49'10.5220"W, MZUCR- INB0001494593, 1 valve (J. Espinosa id., Y. Camacho col. 24/v/1998). BRAZIL. **Bahia;** Salvador, Todos os Santos bay, MZSP 105725, 1 specimen (CETESB col. ii/2000). **Rio de Janeiro;** Angra dos Reis, Ilha Grande bay, 7.5 m depth, MZSP 22747, 9 specimens (Emília sta. 61, Ilha Grande Project, 10/xii/1965); 9.6m depth, MZSP 22780, 4 specimens, (Ilha Grande Project, Penna- Neme col. 25/vii/1966); Sepetiba bay, Itacuruça Island, MNRJ 12458, 8 specimens. **Santa Catarina;** Bombinhas, 3–5 m depth, CENEMAR, 2 specimens, (in shrimp net, J. Tarasconi col., 03/iv/ 1994); 5-10 m depth, CMAC, 3 valves (J. Tarasconi col, 1995); 8–10 m depth, CMAC, 2 valves (trawled by shrimpers, J. Tarasconi col. x/1994).

## Discussion

Below, characters described on this study are compared between classical anatomical studies on genus *Diplodonta*. Lastly, general aspects concerning the species are analyzed, improving current data aboutthe genus.

**Shell:** The original description by d’Orbigny (1846) lacks data on the shell variation, despite its noteworthy emphasis on the rounded form. Beyond the overall shape, a typical shell of *Diplodonta portesiana* has other new discovered exclusive features, such as: ligament length ~1/5 of total animal length, with its anterior limit below the umbo; a short and deep nymph. Besides, *Diplodonta portesiana* shell shows a concentric pattern of microscopically concavities. Mikkelsen and Bieler (2007) already demonstrated this character on ungulind shells, showing different patterns between species, being radial or concentric patterns.

**Muscular system:** A first description on the species’ adductor muscles was offered by Mittré (1850), who noticed a slight discrepancy in shape and size of both adductors in a specimen of *Diplodonta punctata*. Allen (1958) justified the presence of an elongated anterior adductor by relating it with the ability to form an anterior inhalant tube. This was the first time that pairs of anterior and posterior foot retractor muscles were described for the genus, including the absence of a pair of protractor muscles in such an active burrowing genus (Domaneschi 1979). A new pair of muscles is described in this paper: the inhalant and exhalant channel muscles. It is possible that this pair of muscles has a role in controlling the contractions of the inhalant and exhalant apertures, as well as in sustaining the muscular wall to compensate the lack of an exhalant siphon.

**Foot:** The foot shape is one of the most outstanding characters in Ungulinidae. It was described by Duvernoy (1842) and Mittré (1850) as being long and cylindrical, with a terminal expansion. In histological studies, Allen (1958) showed differences between muscular fibers of the tip and proximal portions of the foot’s terminal expansion, as well as a large concentration of mucous cells. Other biological considerations further improved this knowledge, especially regarding the production of mucous tubes: Haas (1942) discussed the production of mucous-sediment tubes in *Diplodonta orbella* Gould, 1852 to accommodate the siphons; Domaneschi (1979) described the production of mucus by *Diplodonta punctata*. As no specimen of *Diplodonta portesiana* has been seen alive, we can only infer this habit by some mucous-sand structures surrounding the anterior portion of some shells.

**Mantle:** The current translucent mantle and the pedal gape, common to the genus (Mittré, 1850), was found in *Diplodonta portesiana*. A concentration of glandular cells below the main rejection tract, i.e., between the outer and middle folds of the mantle, and no differences between the papillae of the middle and inner folds have been found in histological studies of ungulinids (Allen 1958).

**Pallial cavity:** Duvernoy (1842) and Mittré (1850) described two demibranchs for each gill, a feature confirmed in this study. The palps are thin (Mittré 1850) and show a moderate size, with a variable folded area. Allen (1958) described the rejection tracts of palps as very narrow and located at their ventral margin, a feature also found. A new character is, moreover, described here: a “ring-like” tissue fused with the muscular and visceral sac surfaces located below the connection of the gill to the visceral sac. It was postulated that this could be analogous to the flower-like organ described for the Galeommatidae (Mikkelsen and Bieler 1992, Simone 2001). But these structures only occur at the anterior portion of the foot, differing from the structure of the ungulinids, which occurs at the posterior half of foot. The gills of *Diplodonta* are free from each other, while other ungulinid genera have their gills fused in their posterior portion by a tissue originated in same location as the “ring-like” tissue described here. We offer here a first hypothesis regarding this “ring-like” tissue: considering the existence of ungulinids with fused and free gills, this “ring-like” structure could be a residual tissue from a complete connection between the gills.

**Circulatory system:** A ventricle and intestine crossing was noticed by Duvernoy (1842). Allen (1958) described the circulatory system as not significantly different from other Eulamellibranchia. Greater attention should be called to the thin and delicate pericardial tissue. The pair of auricles has thin walls and is connected to the central axis of demibranch.

**Digestive system:** The digestive structures are delicate and mostly covered by the gonads and the digestive gland. The most striking character in the digestive system is the stomach, complex, possessing several sorting areas and folds. Allen (1958) described a *Diplodonta* stomach as morphologically and functionally similar to other Eulamellibranchia. Compared to Allen’s results, the number of ducts attached to the gastric wall can be variable between species. Purchon (1960), based on Allen’s description, classified the ungulinids’ stomach as “type V” mainly due to: conjoined style-sac and intestine; major typhlosole penetrating both diverticula; gastric shield penetrating dorsal hood and left pouch openings; ducts to digestive diverticula opening on right wall of stomach; presence of a sorting area on roof of anterior side of dorsal hood; a sorting area inside left pouch and presence of a sorting area below esophagus orifice. *Diplodonta portesiana* stomach shows all characters described above, and is strongly characterized by two main gastric characters: firstly, the constancy in number of lateral ducts of digestive diverticula, each one attached at each side of stomach; second, the number of sorting areas covering gastric chamber, with one starting below esophagus’s rim and a second covering dorsal hood surface, as described to a characteristic ungulinid stomach.

**Central nervous system:** Duvernoy (1842) noticed three ungulinid pairs of ganglia, which were later described by Allen (1958) as having no great modifications when compared to other Eulamellibranchia. This general feature was found in *Diplodonta portesiana*, except for a connection described between the pallial nerve and the anterior adductor nerve, also found in other Lucinoidea.

As stated above, a revision of the genus *Diplodonta* is still in progress, and several diagnostic morphological and shell characters have already been found. This paper, with a more complete description of *Diplodonta portesiana*, is only the first step of this effort. The other species already are under study in order to improve the taxonomy of *Diplodonta* in the near future. The further publications will allow more comparisons and discussion of the taxonomical features, from species to family levels.

## Supplementary Material

XML Treatment for Diplodonta portesiana
